# Piezoresponse, Mechanical, and Electrical Characteristics of Synthetic Spider Silk Nanofibers

**DOI:** 10.3390/nano8080585

**Published:** 2018-08-01

**Authors:** Nader Shehata, Ishac Kandas, Ibrahim Hassounah, Patrik Sobolčiak, Igor Krupa, Miroslav Mrlik, Anton Popelka, Jesse Steadman, Randolph Lewis

**Affiliations:** 1 Department of Engineering Mathematics and Physics, Faculty of Engineering, Alexandria University, Alexandria 21544, Egypt; ishac@vt.edu; 2 Center of Smart Nanotechnology and Photonics (CSNP), SmartCI Research Center, Alexandria University, Alexandria 21544, Egypt; 3 USTAR Bioinnovations Center, Utah State University, Logan, UT 84341, USA; ibrahim.hassounah@gmail.com (I.H.); jessesteadman98@gmail.com (J.S.); randy.lewis@usu.edu (R.L.); 4 Physics Department, Kuwait College of Science and Technology (KCST), Doha District 13133, Kuwait; 5 Center of Advanced Materials, Qatar University, Doha 2713, Qatar; patrik@qu.edu.qa (P.S.); igor.krupa@qu.edu.qa (I.K.); anton.popelka@qu.edu.qa (A.P.); 6 Centre of Polymer Systems, University Institute, Tomas Bata University in Zlin, Nad Ovcirnou 3685, Zlin 76001, Czech Republic; mrlik@utb.cz

**Keywords:** spider silk, sensor, mechanical vibrations, humidity, piezoelectric, nanofibers

## Abstract

This work presents electrospun nanofibers from synthetic spider silk protein, and their application as both a mechanical vibration and humidity sensor. Spider silk solution was synthesized from minor ampullate silk protein (MaSp) and then electrospun into nanofibers with a mean diameter of less than 100 nm. Then, mechanical vibrations were detected through piezoelectric characteristics analysis using a piezo force microscope and a dynamic mechanical analyzer with a voltage probe. The piezoelectric coefficient (*d*_33_) was determined to be 3.62 pC/N. During humidity sensing, both mechanical and electric resistance properties of spider silk nanofibers were evaluated at varying high-level humidity, beyond a relative humidity of 70%. The mechanical characterizations of the nanofibers show promising results, with Young’s modulus and maximum strain of up to 4.32 MPa and 40.90%, respectively. One more interesting feature is the electric resistivity of the spider silk nanofibers, which were observed to be decaying with humidity over time, showing a cyclic effect in both the absence and presence of humidity due to the cyclic shrinkage/expansion of the protein chains. The synthesized nanocomposite can be useful for further biomedical applications, such as nerve cell regrowth and drug delivery.

## 1. Introduction

Recently, spider silk has been used for its unique properties, such as its versatility and remarkable strength, with a specific strength that is 5 times higher than that of steel and 2 times higher than that of Kevlar [1]. Compared to silkworm silk, spider silk is considered the strongest, most elastic, and toughest biomaterial, and it is suitable for widespread applications [2]. It can be applied as films or scaffolds to enhance tissue regeneration in skin, nerve, bone, and cartilage, or to repair ruptured connective tissues, such as tendons and ligaments [3]. Web-weaving spiders use impressive silk, where the proteins’ in the silk can be described as virtually non-diverged [4,5]. In the last three decades, several researchers have studied the mechanical and chemical properties of spider silk for its use as a promising biomaterial for a variety of applications [6,7,8]. The mechanical properties of natural spider silk have received special attention, because spider silk absorbs the energy of a captured insect without breaking and without the insect bouncing away from the web [9]. Different parameters affect the proteins of formed silk, such as temperature, hydration state, protein concentration, and extension rate. Minor ampullate silk protein (MaSp) has a two-protein composition in every orb-weaving spider [10,11,12]. MaSp proteins are composed of GGX and polyalanine repeats at certain positions, with no substitutions. Recent research also revealed a hierarchical structure of the silk fibers, leading to a better understanding of their promising mechanical performance. However, studies are lacking on the formation of silk nanofibers via the electrospinning process, along with the usage of this nanostructure as a multifunction sensor based on its piezoelectric, mechanical, and resistive characteristics. Such research can facilitate the use of spider silk nanostructure for biomedical applications inside in vivo media [13,14,15,16].

An interesting feature of silk proteins is their piezoelectricity. Piezoelectricity is the ability of certain crystalline and liquid crystalline materials that lack a center of symmetry to convert mechanical energy to electrical energy, and vice versa [17]. The shear piezoelectricity of silk materials was the subject of early research [18]. Recently, the shear piezoelectricity of silk obtained from the *Bombyx mori* cocoon was studied [19]. It was found that β-sheet content—which can be influenced by mechanical, chemical, or thermal treatment—plays a predominant role in determining the intrinsic shear piezoelectricity of silk films. This property is important for future applications of spider silk nanofibers as connections between damaged nerve cells during nerve system regrowth.

The contribution of this research is the analysis and application of electrospun nanofibers from synthetic spider silk as a sensor for both mechanical vibration and humidity. In detail, this work introduces the generation of synthetic spider silk through extraction from transgenic goats, followed by the production of nanofibers using an electrospinning technique. In the electrospinning, the process parameters along with spider silk solution properties were optimized to generate the best nanofiber mats with minimum beads. Then, the formed nanofiber mats were analyzed, using both a piezoresponse force microscope (PFM) and a dynamic mechanical analyzer (DMA), at low-frequency mechanical vibrations similar to those created by the impact of nerve cell bridges. Then, the nanofiber mats were utilized as a mechanical vibration sensor using a voltage indicator, and, conversely, as a voltage sensor using a mechanical deformation indicator. Structural analysis was conducted through a scanning electron microscope (SEM) and Fourier transform infrared (FTIR) analysis, which showed the size and formation of beta-sheets that enable the formed nanofibers to act as a piezoelectric mat. For their potential sensing functions, the generated spider silk nanofibers mats were characterized mechanically and electrically under changing humidity conditions. These conditions are analogous to those of a medical host, wherein the spider silk protein nanofibers can be used to connect damaged nerve cells. Therefore, the nanofibers mats can be used as a humidity sensor, especially for relative humidity (RH) levels above 75%, according to mechanical and resistance measurements. 

## 2. Materials and Methods

### 2.1. Chemicals Synthesis

Spider silk proteins were extracted from transgenic goats by Nexia Biotechnologies. As the proteins are not optimized for mammalian expression and contain a C-terminal non-repetitive region with ensured expression in the mammary gland exclusively. The silk proteins (MaSp4) reached levels equivalent to 3–4 g of the protein/L of milk. The proteins can be purified from bacteria using a His-tag, and from milk using modified protocols from Nexia (Vaudreuil-Dorion, QC, Canada) for goat proteins. Spider silk was initially synthesized by dissolving MaSp4 pure protein in a mixture of formic acid (concentration of 88%) and 1,1,1,3,3,3-hexafluoro-2-isopropanol (HFIP) with a ratio of 1:1, both from Alfa Aesar (Tewksbury, MA, United States). A few droplets of Triton X-100, supplied by Sigma Aldrich (St. Louis, MO, USA), were added as a surfactant.

### 2.2. Electrospinning Process

**Figure 1:**
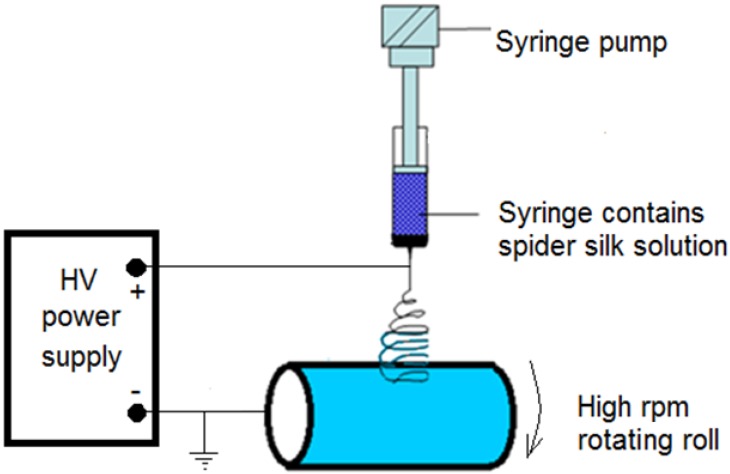
Simple schematic diagram of the electrospinning setup used to generate spider silk nanofiber mats.

The electrospinning unit (IME Technologies, Eindhoven, The Netherlands) consists of a high-voltage power supply and a syringe pump, which is used to regulate the pumping rate of the polymer solution. Then, the solution was pumped with a 5 mL plastic syringe along with an 18-gauge metallic needle. The target of the electrospinning setup was a rotating metallic collector with a radius of 10 cm covered with aluminum foil. A schematic of the electrospinning setup is shown in Figure 1. The voltage power supply was connected to the needle, while the collector was grounded, with a separation distance of 15 cm. The voltage difference between the needle and the target was 25 kV, with a 1 mL/h flow rate for the polymer solution and 1 h running time per sample. The speed of the roll or collector was accelerated up to 1500 rpm to obtain the electrospun nanofibers.

### 2.3. Characterization Procedures and Sensing Analysis

The morphological features of the fibers were examined by a field emission scanning electron microscope (FEI Quanta 200, Graz, Switzerland). Fourier transform infrared (FTIR) spectrometry was used to identify the chemical groups of the prepared electrospun mats. FTIR data were recorded on an FTIR spectrometer Spectrum 400 (Perkin Elmer, Akron, OH, USA). 

In mechanical vibration sensing, the dynamic forcing was provided by the isolated clamps of a dynamic mechanical analyzer (DMA), model RSA-G2 (TA Instruments, Detroit, MI, USA). The frequency of the applied force was well below that of the fundamental resonance frequency of the system, which was formed by the elastic polypropylene/polyvinylidenefluoride (PP/PVDF) sample and the seismic mass. In the tests described here, the forcing frequencies were in the 10–100 Hz range, while the resonant frequency of the system was well above 10 kHz. To isolate the frequencies of interest for the tests, mass values were calculated from various applied normal forces, using spurious vibration modes in the DMA and other components. All masses had the same contact area of 12.56 cm^2^.

In the piezoelectric analysis, a lumped parameter model of a piezoelectric device in the 3-3 mode shunted by a load resistance was obtained by Equation (1) [20]:

(1) $$Fd_{33}=Cv+\frac{v}{R},$$

where *F* is the force on the device in the poling direction, *d*_33_ is the electromechanical coupling constant, *C* is the static capacitance of the device, *v* is the voltage across the electrodes, *R* is the load resistance, and a dot represents the derivative with respect to time. With respect to the applied mechanical force (*F*), the variable force with time was calculated by Equation (2), as follows:

(2) F(t)=ma(t),

where *m* is the effective mass calculated from Equation (2), and *a*(*t*) is the vertical acceleration of the mechanical analysis. This model is applicable when the frequency of the applied force is well below that of the fundamental resonance frequency of the system formed by the sample. Using both Equations (1) and (2), one can obtain the following frequency response function, which relates the electrode voltage output (*V*) to the base acceleration input (*A*).

(3) $$\frac{V}{A}=\frac{jRmd_{33}\Omega }{jRC\Omega +1}.$$

To extract values of *d*_33_, harmonic acceleration frequency (Ω) sweep tests were conducted with various load resistances. In the case of low frequencies, *RC*Ω << 1, yielding experimental frequency response functions that are approximately linear, i.e.,

(4) $$\left|\frac{V}{A}\right|\approx Rmd_{33}\Omega .$$

The piezoresponse force microscopy (PFM) technique was used to analyze the piezoresponsive properties of the prepared samples. The atomic force microscopy (AFM) system MFP-3D (Asylum Research, High Wycombe, UK) was employed for this purpose, using the single-frequency PFM mode. A conductive tip with platinum-deposited cantilever AC240TM (Olympus, Tokyo, Japan), with a 2 N/m spring constant and 70 kHz resonance frequency, was chosen for these analyses. The tip was first calibrated using thermal GetRealTM mode to obtain an exact value of the spring constant and accurately convert the raw signal (in V) to pm. The measurements were carried out in contact mode by applying voltage to the AFM tip, varied from 1 V up to 10 V, and the subsequent amplitude response was recorded. The obtained data were then evaluated using Igor Pro 6.37 software (WaveMetrics, Portland, OR, USA).

To correlate between mechanical properties and humidity, the mechanical properties of the formed nanocomposite were analyzed using MTS Synergie 100 (Eden Prairie, MN, USA). The sample was shaped as a yarn with a small diameter (in the range of 300–700 µm) after using twisted mats (with a size of about 1 × 12 cm). The mats were twisted by a fringe twister (Lacis cord maker and fringe twister, Berkeley, CA, USA). Then, they were glued to a C-shaped plastic card. The sample was held by two clamps. The software of MTS TestSuite (MTS, Eden Prairie, MN, USA) controlled the tension force and calculated the stress and strain, then drew the well-known stress–strain curve. Then, calculations were performed to get all required characteristics.

**Figure 2:**
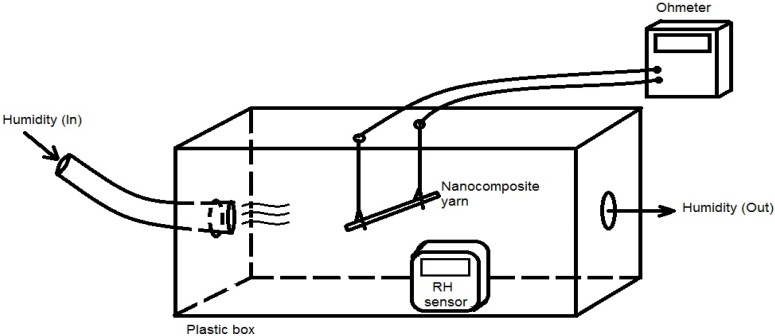
Schematic diagram of the resistance measurement setup, with humidity produced with a commercial humidifier.

The electric resistance properties of the designed nanocomposite, at different RH values, were determined through a simple setup, as shown in Figure 2. The nanocomposite was twisted into yarns with diameters 500–700 µm and with a length of approximately 4 cm. Each end of the yarn was attached to a metal clamp connected to a commercial multimeter (Fluke, Everett, WA, USA). The controlled environment was then exposed to a particular relative humidity level, and the spider silk’s resistance was then recorded as a function of time and humidity. The resistance value was generally recorded for 30 min per experiment. A relative humidity (RH) level of 99% was reached to provide a fully humid environment to the prepared nanocomposite.

## 3. Results and Discussions

### 3.1. Characterization of Spider Silk Nanofiber Mats

**Figure 3:**
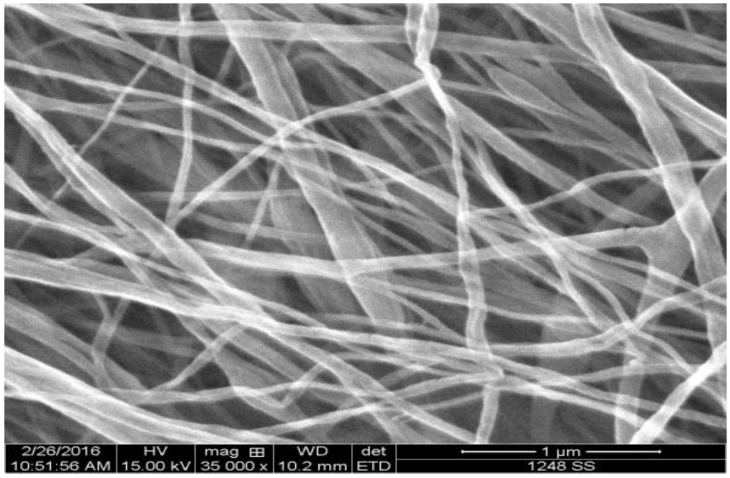
Scanning electron microscope (SEM) image of spider silk nanofibers, with mean diameter ~70 nm.

**Figure 4:**
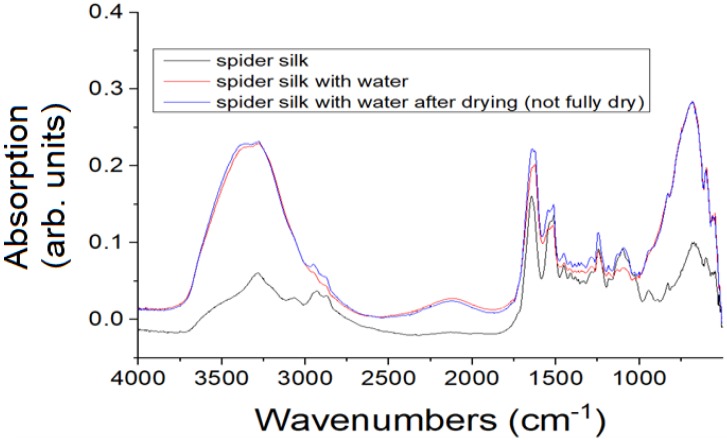
FTIR spectrum of spider silk nanofibers showing the formed chemical bonds corresponding to beta-sheets, with increased number of peaks due to the humid environment.

An SEM image of a spider silk nanofiber mat is shown in Figure 3. The mean diameter of spider silk nanofibers was found to be ~70 nm, which is one of the smallest spider silk nanofibers in the literature. The synthesized nanofibers were analyzed using Fourier transform infrared (FTIR) spectrometry to identify the chemical groups of the prepared electrospun mats. Figure 4 shows the FTIR spectroscopy pattern of the spider silk electrospun nanofibers. FTIR analysis was used to confirm the existence of MaSp1 and to observe any peak shifts that characterize the amide I, amide II, and amide III bands. It can be observed that the main chemical groups in spider silk, such as primary and secondary amides, are located in the range 1470–1700 cm^−1^. However, the range 1160–1320 cm^−^^1^ shows the peaks of different secondary structures (alpha helix at 1182 cm^−1^, random coil at 1245 cm^−1^, and beta-sheets at 1287 cm^−1^). In the case of the wet samples, the formed chemical bonds corresponding to beta-sheets have an increased number of peaks due to the humid environment.

### 3.2. Mechanical Vibration Sensing

**Figure 5:**
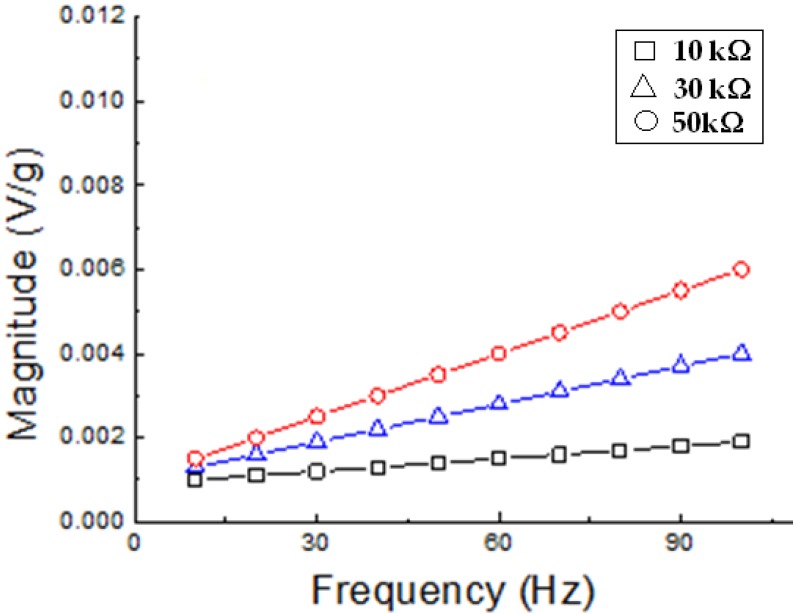
Frequency dependence of the investigated electromechanical properties upon dynamic stimulation for various values of loads and different samples.

Using the DMA to initiate vibrations, the spider silk mat produced electric voltages, as shown in Figure 5, at different load resistance. The obtained voltage signal was enhanced using increasing load resistance. Also, by increasing the frequency of vibrations, the spider silk nanofiber mat showed a linear response in the collected voltage signal. The extracted voltage increased with increasing applied frequency, whatever the load resistance. The mat of electrospun spider silk nanofibers was analyzed to extract its electromechanical coupling coefficient (*d*_33_). The *d*_33_ coefficients were measured and calculated according to the procedure already described in the experimental section, and the values are shown in Table 1. The average value of *d*_33_ coefficient is found to be 3.62 pC/N. It is noted that load resistance has a nearly negligible effect on the final values of the *d*_33_ coefficient, while the nature of the material itself plays a dominant role.

**Figure 6:**
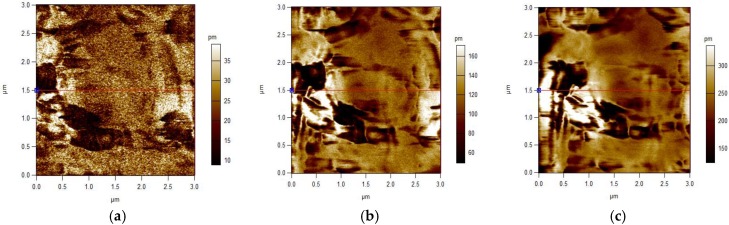
Atomic force microscopy (AFM) surface morphologies at different applied voltages (**a**) 1 V, (**b**) 5 V, and (**c**) 10 V.

**Figure 7:**
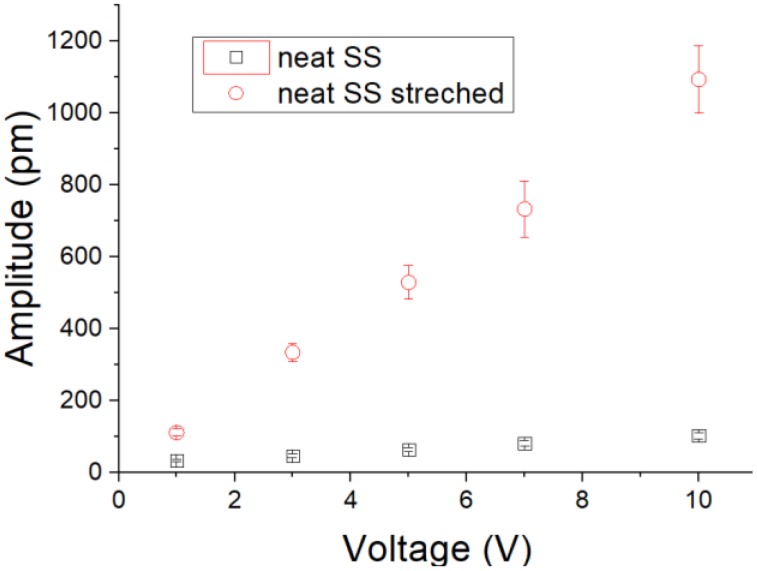
Mechanical deformation amplitude at different applied voltages for spider silk mats under normal conditions and under mechanical compression.

Also, the applied voltage can be detected from the very sensitive mechanical deformation. From the PFM analysis, our synthesized spider silk nanofiber mats were subjected to different applied electric potentials. Some examples of mechanical deformation of the mats, as detected by AFM, are shown in Figure 6. It can be observed that by increasing the applied electric potential, the formed diploes inside the mats were more stretched (longer) and caused a higher mechanical deformation amplitude. The detected deformation amplitude, along with different applied voltages, is shown graphically in Figure 7, both with and without applied compression. The compressed spider silk mat became very responsive to the applied voltage. In other words, the polarized diploes inside the nanospider chains were compressed and stretched along longer paths when the fibers were compressed.

### 3.3. Humidity Sensing through Mechanical Properties

**Figure 8:**
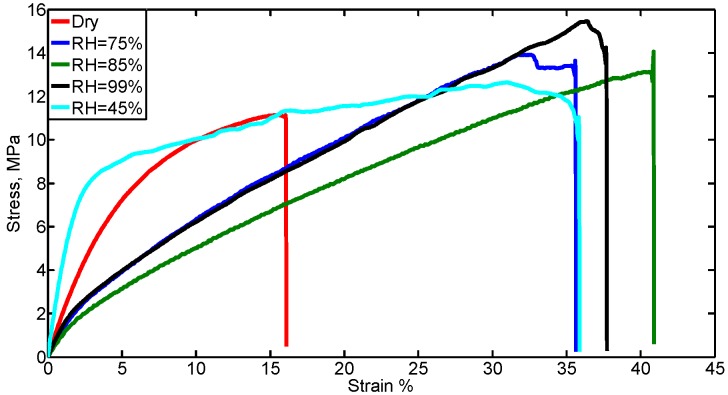
Stress–strain curve for spider silk (SS) nanofibers at different relative humidity (RH) values. A clear change in the strain range and ultimate stress is observed at all high-level humidity, compared to the case of the dry spider silk nanofiber mat.

**Figure 9:**
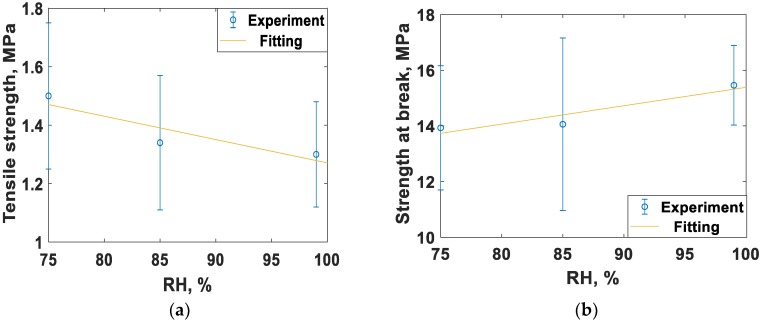

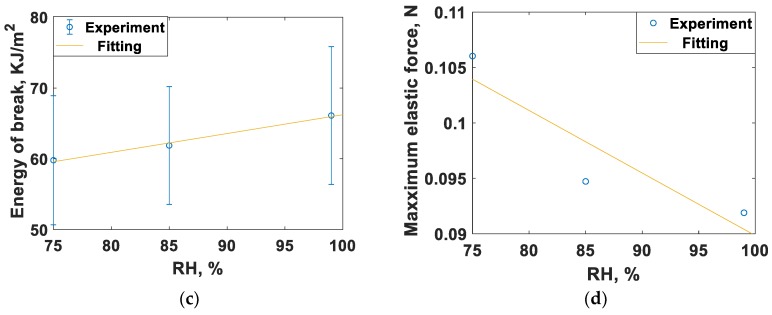
Change of mechanical properties at relatively high levels of humidity. The mechanical properties are (**a**) tensile strength, (**b**) strength at break, (**c**) energy of break, and (**d**) maximum elastic force.

The mechanical properties of dry (RH ~ 16%) spider silk nanofibers and different levels of RH are shown in Figure 8. As shown in Figure 8 and Table 2, the elastic modulus and tensile strength were reduced, while the maximum strain, strength at break, and energy of break increased when the humidity of the surrounding medium is increased. Table 2 summarizes the comparison between spider silk at normal humidity (dry) and at different RH values. For RH values 75%, 85%, and 99%, the same trend was observed, i.e., a decrease in the elastic modulus and tensile strength, but an increase in maximum strain, strength at break, and energy of break. For an RH of 45%, all properties were increased. Also, fluctuations in some of the mechanical properties values were noticed when the RH value increased. This can be explained as follows. At low RH values, the electrospun nanofiber is still well integrated and can be handled easily. However, at high RH, the nanofiber sample becomes cotton-like in texture, indicating poor bonding, which agrees with results in [21]. For the synthesized spider silk protein nanofibers and for the shown RH values, the critical value was 45%. Figure 9a–d show the linear variation of some mechanical characteristics with higher levels of RH, above 70%. The sensitivity of the mechanical properties at levels of RH above 70% was −5.6 × 10^−4^ N for each 1% change of RH. At RH levels below 70%, these properties showed some odd behavior, thus, spider silk nanofibers mats are recommended to sense levels of humidity above 70%, which is suitable for biomedical applications in semi-aqueous media, such as that used for nerve system regrowth.

### 3.4. Resistivity Measurements versus Humidity

**Figure 10:**
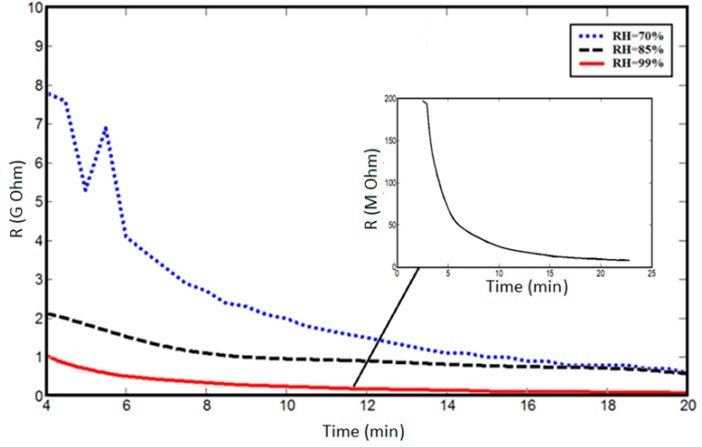
Resistance decay at different RH values (inset graph is the detailed resistance decay at full humidity). In all RH cases, the resistance starts to be saturated after nearly 20 min of continuous exposure to humidity.

**Figure 11:**
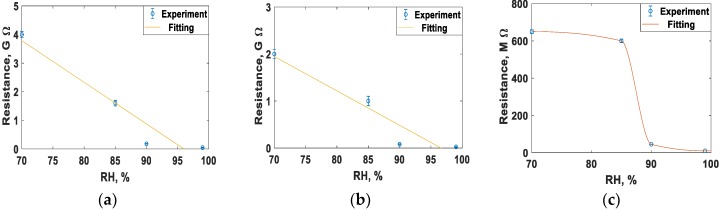
Resistance change with variable RH at different times of humidity exposure: (**a**) 6 min, (**b**) 10 min, and (**c**) 20 min.

As with the mechanical characteristics, the resistance of spider silk yarns is correlated to RH, when RH values are above 70%. In the dry environment or at RH values below 70%, there were no detected resistances for the spider silk nanofiber yarns for the same measurement time of 30 min. However, the yarns started generating measurable or readable resistance after exposure to a humid environment of 70% RH. Beyond this level of RH, the resistance values were in the measuring range of the multimeter, and we observed an exponential-like decay in the resistance of the spider silk nanofiber, as shown in the inset of Figure 10. In the first few minutes, the readings were above the maximum readable range of the multimeter, and later the values started to decay to below the readable range, as shown in Figure 10, which shows the resistance decay at different RH values. By increasing the RH value, the resistance values started to decay to smaller values for the same humidity exposure time. In all cases, the resistance started to show saturation after a humidity time close to 20 min. Then, RH was detected according to the measured values of resistance, but it was preferred to perform measurements after the saturation of resistance to allow sufficient humidity exposure time, as shown in Figure 11. The relation between resistance and RH is relatively more linear due to a lower time of humidity exposure, as shown in Figure 11a, with slope of −0.5 × 10^8^ Ohm for each 1% change of RH. At higher humidity exposure times, as shown in Figure 11c, there is a clear difference in behavior and values of the resistances at RH below 90% compared to levels of RH beyond 90%, until achieving the fully humid environment, “RH ~ 99%”. There is a tradeoff between the exposure time of humidity, linear behavior of resistance, and the range of measured values. At low exposure times, the resistance change with humidity is more linear, but with measured resistance in the gigaohm range. On the other hand, lower values in the Mega Ohm range could be measured at higher exposure times, but the linearity between the resistance and RH was lost.

**Figure 12:**
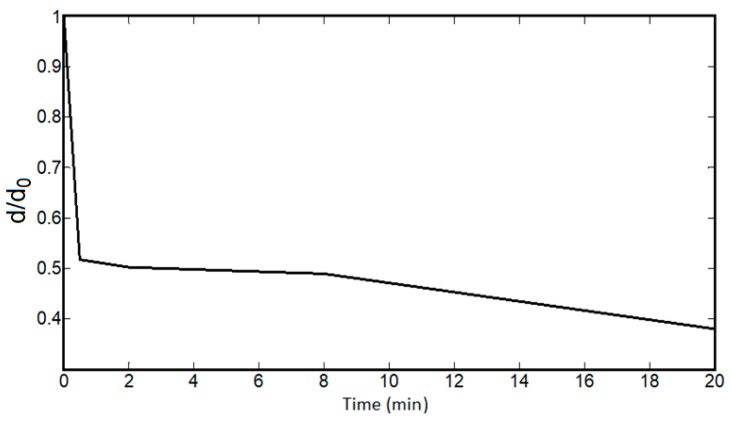
Sharp decay of the diameter of spider silk nanofiber yarns versus time. In the first minute of humidity exposure, the diameter is dramatically decreased.

**Figure 13:**
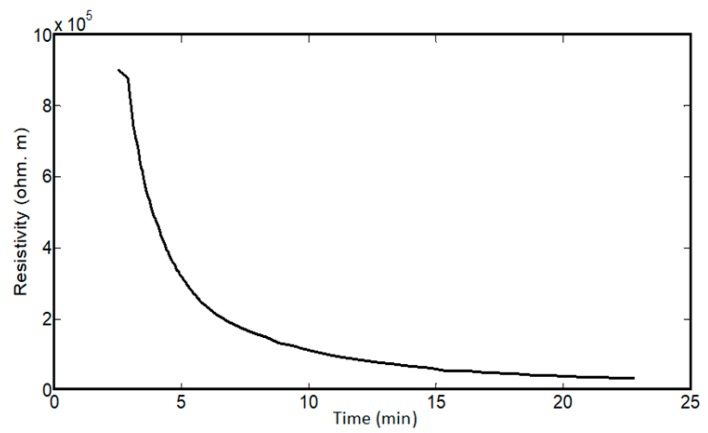
Decay of electric resistivity of spider silk nanofibers versus time in a fully humid environment.

The impact of humidity on spider silk can be explained by the resistance decay mainly resulting from the shrinkage of the spider silk proteins, and not from external diameter reduction. To check the effect of dimensional parameters, the external diameter of the spider silk nanofiber yarn was measured in a humid environment over time, with the yarn length was kept constant by fixing the distance between the two clamps holding the yarn. As shown in Figure 12, the diameter quickly decayed with time and was saturated after 2–3 min. This proves that the saturation value of the diameter over the resistance decay period—and, consequently, the resistance decay—is mainly based on the internal resistivity decay with no dependence on external dimension variation. Figure 13 shows the resistivity decay of spider silk yarns in a fully humid environment as function of time.

**Figure 14:**
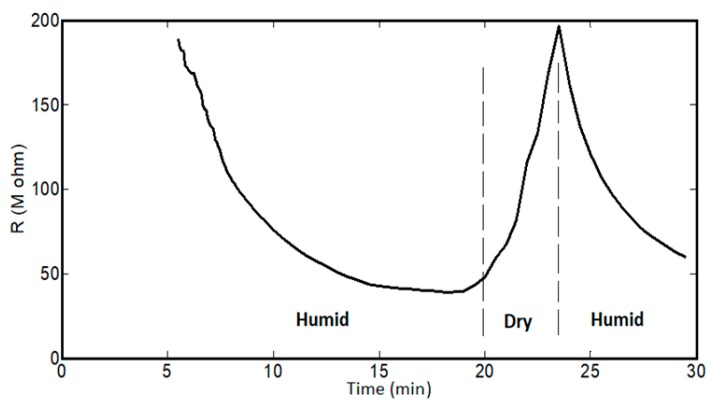
Cyclic resistance of the nanocomposite at RH levels of 90% and 16% (“normal condition”).

Regarding the electric resistance at variable humidity conditions, when the humidity started to be reduced to normal relative humidity, which was 16% in our lab, the resistance was elevated again. When the humidity was then returned to the 99% level, the resistance dropped again. Therefore, the resistance of spider silk nanofibers shows a cyclic effect with changing humidity conditions (between dry and wet conditions), as shown in Figure 14. Both length and diameter of the yarn were found to be approximately constant for both dry conditions and wet humidity. Therefore, the cyclic effect is not related to changes in external dimensions, but it is related to cyclic changes in internal resistivity due to the cyclic shrinkage/expansion of spider silk proteins according to the periodic dry/wet conditions. This cyclic feature is important for using the spider silk nanofibers as a multi-usage sensing unit, not only as a single-use unit.

## 4. Conclusions

This work demonstrates the ability to generate piezoelectric synthetic spider silk nanofibers using an electrospinning technique, and their application as both a vibration and humidity sensor. Piezoelectric characteristics were analyzed using both a piezo force microscope and dynamic mechanical analyzer with a voltage probe. The piezoelectric coefficient (*d*_33_) was measured to be 3.62 pC/N. In addition, there was increased mechanical deformation of compressed mats at higher applied voltage levels. This work describes the fabrication of relatively small (nanosized) and elastic spider silk nanofibers with an average diameter of less than 100 nm, an elastic modulus of 4.32 MPa, and a maximum strain of up to 40.90%, with linearly changing mechanical characteristics at high ranges of RH beyond 70%. Also, the high range of RH—above 70%—can be detected through the electric resistance, with a sensitivity of 0.15 Giga Ohm per 1% change of RH. The electric resistance shows a cyclic behavior when the humidity is removed. This work can be used develop potential biomedical applications of electrospun spider silk nanofibers, such as their use as bridges between damaged nerve cells.

## Figures and Tables

**Table 1:** Values of the *d*_33_ coefficients (pC/N) for electrospun spider silk nanofibers.

Load Resistance	10 kΩ	30 kΩ	50 kΩ
*d*_33_	3.67	3.66	3.53

**Table 2:** Comparison of mechanical properties of SS nanofibers in different RH conditions.

Mechanical Properties	Dry	RH = 45%	RH = 75%	RH = 85%	RH = 99%
Elastic Modulus (MPa)	1.89 ± 0.30	4.32 ± 0.77	1.24 ± 0.32	0.97 ± 0.16	1.45 ± 0.23
Tensile strength (MPa)	3.57 ± 0.5	4.47 ± 0.6	1.50 ± 0.25	1.34 ± 0.23	1.30 ± 0.18
Maximum strain (%)	16.1 ± 4.2	35.88 ± 7.11	35.65 ± 3.58	40.90 ± 6.92	37.74 ± 3.52
Strength at break (MPa)	11.13 ± 1.88	12.64 ± 2.13	13.93 ± 2.23	14.06 ± 3.1	15.46 ± 1.43
Energy of break (kJ/m^2^)	24.67 ± 7.97	72.52 ± 9.67	59.8 ± 9.12	61.88 ± 8.31	66.11 ± 9.73
